# miR-16-5p enhances sensitivity to RG7388 through targeting *PPM1D* expression (WIP1) in Childhood Acute Lymphoblastic Leukemia

**DOI:** 10.20517/cdr.2022.113

**Published:** 2023-04-29

**Authors:** Maryam Zanjirband, Soheila Rahgozar, Narges Aberuyi

**Affiliations:** Department of Cell and Molecular Biology & Microbiology, Faculty of Biological Science and Technology, University of Isfahan, Isfahan 15100, Iran.

**Keywords:** Pediatric ALL, miR-16-5p, RG7388, *PPM1D*, p53

## Abstract

**Aim:** Given the encouraging results of the p53-Mdm2 inhibitor RG7388 in clinical trials and the vital function of miR-16-5p in suppressing cell proliferation, the aim of the present study was to investigate the combined impact of RG7388 and miR-16-5p overexpression on the childhood acute lymphoblastic leukemia (chALL).

**Methods:** miRTarBase and miRDB, along with KEGG and STRING databases, were used to predict miR-16-5p target genes and explore protein-protein interaction networks, respectively. B- and T-lymphoblastic cell lines, in addition to patient primary cells, were treated with RG7388. Ectopic overexpression of miR-16-5p in Nalm6 cell line was induced through cell electroporation and transfection of microRNA mimics was confirmed by qRT-PCR. Cell viability was evaluated using the MTT assay. Western blot analyses were performed to evaluate the effects of RG7388 and miR-16-5p upregulation on the protein levels of p53 and its downstream target genes in chALL cells. Paired sample t-test was employed for statistical analyses.

**Results:** MTT assay showed RG7388-induced cytotoxicity in wild-type p53 Nalm6 cell line and p53 functional patient primary cells. However, CCRF-CEM and p53 non-functional leukemic cells indicated drug resistance. Western blot analyses validated the bioinformatics results, confirming the downregulation of WIP1, p53 stabilization, as well as overexpression of p21^WAF1^ and Mdm2 proteins in Nalm6 cells transfected with miR-16-5p. Moreover, enhanced sensitivity to RG7388 was observed in the transfected cells.

**Conclusion:** This is the first study indicating the mechanistic importance of miR-16-5p overexpression in chALL and its inhibitory role in leukemia treatment when combined with the p53-Mdm2 antagonist, RG7388. These findings might be useful for researchers and clinicians to pave the way for better management of chALL.

## INTRODUCTION

Acute lymphoblastic leukemia (ALL) is one of the most common blood cancers in children with a low incidence rate of *TP53* mutations at diagnosis, which highlights this type of malignancy as an attractive candidate for treatment with p53-Mdm2 antagonists. Although 85%-90% of patients respond to the treatment, there are subsets that are refractory to the therapy and relapse is prevalent amongst individuals who achieve complete remission^[1,2]^. For this reason, new strategies and treatments are necessary to overcome drug resistance.

Cancer treatment has been recently improving with the introduction of targeted therapies to achieve greater specificity and less cytotoxicity. Owing to the main function of p53 in the enhancement of cell cycle arrest, response to DNA repair and apoptosis, enormous efforts have been made to advance new cancer treatments based on p53-targeted therapy. p21 is a well-known determinant of cell cycle arrest, which increases following induction of p53 stabilization and its activity^[3]^. The incidence rate of *TP53* mutations at diagnosis is low in various types of blood cancer including ALL (5%-10%). Therefore, activation of the p53 pathway by non-genotoxic inhibitors of mouse double minute 2 homolog (Mdm2) is a promising strategy to improve cancer therapy in hematological malignancies, including ALL^[4]^. Recently, small molecule inhibitors of p53-Mdm2 interaction have been developed and entered into early-phase clinical trials for the treatment of diverse types of cancer comprising blood cancer^[5-8]^. Amongst those entered into clinical trials, RG7388 has passed phase II clinical trial^[8]^. Thus, the prediction of sensitivity to Mdm2 inhibitors and identification of mechanisms of resistance toward Mdm2 inhibitors would be helpful in stratifying patients who might benefit from these therapeutic agents.

p53 levels and its activity are regulated under a complex network of proteins and microRNAs (miRNAs) to maintain normal levels and proper function of p53. The oncogene Protein Phosphatase, Mg2+/Mn2+ Dependent 1D (*PPM1D*), known as wild-type p53-induced phosphatase 1 (*WIP1*), negatively regulates p53 through different mechanisms, including direct dephosphorylation of p53 and its subsequent inactivation, and dephosphorylation of other proteins involved in the regulation of p53 such as Mdm2 and p53 activating kinases^[9]^. Consistent with the inhibitory effect of Wip1 on the p53 pathway, *PPM1D* gene is amplified and overexpressed in different cancer types including leukemia, suggesting that Wip1 may be a potential therapeutic target for leukemia^[10]^. Recent studies have demonstrated that selective inhibition of Wip1 leads to increased DNA damage response and sensitivity to anti-cancer agents working through the p53 pathway^[10-13]^.

microRNAs are a class of small RNAs ,with an average of 22 nucleotides in length, which interact with the partially complementary sequences in the 3′ untranslated region (3′ UTR) of the target mRNA and negatively regulate its expression^[9,14]^. Although not proven in childhood ALL, miR-16-5p appears to be a major negative regulator of Wip1 protein expression in some cancers, which affects p53-Wip1 feedback loop^[13]^ and can regulate cell fate^[12]^. miR-16-5p interacts with the 3′-UTR binding site of the human *PPM1D* gene and directly represses the Wip1 protein expression, a negative regulator of p53, thereby indirectly promoting p53 activity and its pathway^[13]^. Previous research showed that miR-16-5p feedback loop with p53 and Wip1 increases sensitivity to doxorubicin^[13]^ and affects cell fate determination^[12]^. Moreover, aberrant expression of miR-16-5p has been reported in chronic lymphocytic leukemia (CLL)^[15,16]^.

In the present study, it was hypothesized that cell lines and primary samples harboring functional p53 are more sensitive to RG7388 compared to those with dysfunctional p53, and ectopic overexpression of miR-16-5p in cells with functional p53 affects WIP1 mRNA levels, and consequently respond to the p53-Mdm2 antagonist RG7388 in a p53-dependent manner.

## METHODS

### miR-16-5p-target interaction databases and visualization of protein-protein interaction network

miRTarBase and miRDB validated microRNA-target interactions databases were employed to clarify the interaction between miR-16-5p and PPM1D expression^[17,18]^. The Kyoto Encyclopedia of Genes and Genomes database (KEGG) (http://www.genome.jp/kegg/) was used as a pathway database to find the p53 pathway map (map04115)^[19]^. To visualize protein-protein interaction network between the Wip1 and other proteins with strong confidence (0.700 interaction score), the STRING database (https://string-db.org/) was used^[20]^.

### Chemicals and reagents

The small-molecule Mdm2 inhibitor RG7388 (Idasanutlin) was purchased from SelleckChem (Cambridge, UK). RG7388 was dissolved in dimethyl sulfoxide (DMSO) to provide a 10 mM stock solution and stored in small aliquots at -20 °C. miR-CURY LNA™ Universal RT microRNA PCR kit and miR-CURY LNA™ miRNA Mimics (HAS-MIR- 16 -5p & NEGATIVE CONTROL 5 MIRCURY LNA) were purchased from QIAGEN (Hilden, Germany).

### Cell lines

The leukemic cell lines Nalm6 and CCRF-CEM were sourced from the Pasteur Institute (Iran) authenticated cell bank. The cells were cultured in RPMI-1640 (Gibco, USA) supplemented with 10% (v/v) FBS (Gibco, USA) and 1% (v/v) penicillin/streptomycin and grown at 37 °C, 5% CO2 in a humidified atmosphere. The *TP53* status of Nalm6 cell line is wild-type (NALM6 ATCC CRL-3273™) and the CCRF-CEM cell line (CCRF-CEM ATCC CCL-119™) harbors heterozygous *TP53* mutations (c.524G>A; p.R175H, and c.743G>A; p.R248Q).

### Patients, sampling and cell isolation

Peripheral blood or bone marrow samples (*n* = 10, 5 females and 5 males) from childhood ALL patients were collected. ALL in these patients was clinically diagnosed and pathologically confirmed by a clinical team through phenotypic, immunologic and cytogenetic techniques in the pediatric department of Sayed-ol-Shohada Hospital (Isfahan, Iran). Informed written consent was obtained in accordance with the Declaration of Helsinki, and with approval from the Ethics Committee of the University of Isfahan (ethics agreement number IR.UI.REC.1397.145). Informed written consent was obtained from the children’s parents prior to participation in the study.

2-5 mL of heparinized bone marrow sample or peripheral blood were collected from patients and sent to the Cellular and Molecular Biology Laboratory of the University of Isfahan on ice. Mononuclear cells were extracted and isolated using density gradient Lymphoprep (Axis-Shailed Diagnostics Ltd, Oslo, Norway) according to the manufacturer's protocol.

### Ex vivo cytotoxicity assay

Cytotoxicity of RG7388 was assessed using MTT cell proliferation kit (Alban, Austria). Growth curves were constructed for Nalm6 and CCRF-CEM cell lines (GraphPad Prism statistical analysis software version 8.) to measure cell doubling time and the optimum seeding density for a subsequent growth inhibition assay. Nalm6 (5 × 10^5^/mL) and CCRF-CEM (4.5 × 10^4^/mL) in 100 μL of medium per well of a 96-well plate were treated with a range of concentrations of RG7388 for 96 and 72 h, respectively (according to their cell doubling time). Then, 10 μL of MTT solution was added into the wells. 100 μL DMSO was added after 3 h to dissolve formazan crystals, and absorbance was measured using a stat fax 2000 microplate reader (Awareness Technology, Inc) at 492 nm wavelength. The LC_50_ values, the required concentration of each compound expected to kill 50% of the population, were determined using the statistical software mentioned above.

For patients` samples, primary cells (2 × 10^6^/mL) in 100 μL of medium per well of a 96-well plate were exposed to 0.5% DMSO or 0.5 µM RG7388 (2 × LC_50_ concentration for Nalm6) for 72 h. This concentration was used since sensitive cells respond to it, and the sensitivity is not due to off-target effects. The proportion of the viable cells was measured by comparison between the absorbance of cells exposed to DMSO, as a control, or RG7388, and calculated using the following formula: (%) = [100 × (sample absorbance)/ (control absorbance)].

### Functional assessment of the p53 pathway

To determine the functional status of p53 in ALL patients’ samples (those with enough amount of protein lysates), the modulation of p53 and its transcriptional target gene protein products including Mdm2 and p21^WAF1^ were evaluated following short-term exposure to Mdm2-p53 antagonist RG7388^[21,22]^.

### Western blotting

Nalm6 and CCRF-CEM (2.5 × 10^5^/mL) were seeded in 2 mL culture media per well of a 6-well plate and treated with 0.5% DMSO and a range of concentrations of RG7388. Primary cells (0.5 × 10^6^/mL) were also seeded in 2 mL culture media per well of a 6-well plate and exposed to 0.5% DMSO or 0.5 µM RG7388 (2 × LC_50_ concentration for Nalm6). Cells were harvested and lysed at 6 h. Lysis buffer (12.5 mL Tris HCL, 2 g SDS, 10 mL Glycerol, 67.5 mL Distilled Water) was applied to harvest the whole-cell lysates, followed by sonication. Bradford solutions (100 MG Coomassie Blue 250 G, 50 mL ethanol 96%, 100 mL ortho-phosphoric acid 85% and bringing volume to 1000 mL by adding distilled H_2_O) were used to estimate the concentration of protein in the cell lysates utilizing NanoDrop ND-1000 Spectrophotometer (Thermo Fisher Scientific, USA).

Hand-poured gradient gels were prepared using Bio-Rad mini gel casting apparatus, and two different acrylamide solutions were applied to separate proteins^[23]^. The separated proteins were transferred by perpendicular electrophoresis to a nitrocellulose Hybond^TM^ C membrane (Amersham, Buckinghamshire, UK). Monoclonal mouse anti-human primary antibodies Actin 1:250 (#: C4: sc-47778, Santacruz Biotechnology, INC.), Mdm2 1:300 (#: OP46-100UG, Merck Millipore), p21 1:100 (#: OP64, Calbiochem), p53 1:250 (#: 2B2.68: sc-71817, Santacruz Biotechnology, INC.) and Wip1 1:200 (#: F-10: sc-376257, Santacruz Biotechnology, INC.) were used. Secondary goat anti-mouse HRP-conjugated antibodies (#: P0447/P0448, Dako) were applied at 1:1000. 5% milk/1XTBS-Tween (w/v) was used in order to dilute all antibodies. Enhanced chemiluminescence (GE Life Sciences, UK) and X-ray film (Fujifilm, India) were employed to visualize the proteins. Image J software (National Institute of Health, USA) was used to quantify and analyze the intensity of visualized bands, and the results were normalized to DMSO control.

### Cell transfection

Harvested Nalm6 cells in the exponential growth phase were resuspended in RPMI-1640 (Gibco, USA) supplemented with 0.5% (v/v) FBS (Gibco, USA). 200 µL cell suspension containing 2.5 × 10^6^ of cells were transferred into sterile electroporation cuvettes (0.2 cm gap, Bio-Rad, USA) separately (miR-16-5p mimic and negative control). miR-16-5p mimic and negative control (200 nm) (miR-CURY LNA™ miRNA Mimics, Qiagen) were separately added to the cuvettes in the hood just before electroporation, and the cuvettes gently swirled. Then, the cuvette was placed in the holder in the electroporation system (Eppendorf Multiporator®, Germany) at room temperature. Electroporation was performed in accordance with Multiporator® Transfection Protocol with minor changes [Voltage: 250 V, Time constant (τ): 40 μs, No. of pulses (n): 1] in order to specifically optimize protocol based on the cell type and genetic modifications. After the pulse, the cell suspension was allowed to stand in the cuvette for 5 to 10 minutes at room temperature. Finally, the cell suspension was transferred from the cuvette to 2 mL normal medium in a well of a 6-well plate and incubated for 48 h. In order to determine the impact of overexpression of miR-16-5p on sensitivity to RG7388, 48 h transfected cells were exposed to RG7388 (0.5 µM) for 6 h. Transfected cells were harvested to evaluate cell viability and silencing assessment.

### RNA extraction and cDNA synthesis

Total RNA including preserved miRNAs was extracted from Nalm6 cells and primary samples using TRizol reagents (Invitrogen, California, CA) as per the manufacturer's recommendations. The quality of the RNA and its concentration was assessed with a NanoDrop ND-1000 Spectrophotometer (Thermo Fisher Scientific, USA) by the ratio of 260nm:280nm. The complementary DNA (cDNA) for miR-16-5p was synthesized on 200 ng of total RNA using miR-CURY LNA^TM^ microRNA PCR kit (QIAGEN, Germany) and the thermal cycler (GeneAmp PCR Systems, AB Applied Biosystems) according to the manufacturer’s guidelines.

### Quantitative RT-PCR (qRT-PCR)

Ectopic expression of miR-16-5p was confirmed by real-time PCR assay. qRT-PCR for miR-16-5p converted to cDNA was carried out using the miRCURY LNA SYBR Green PCR kit (Qiagen, Germany), with 50 ng/μL of the cDNA samples per 10 μL final reaction volume, on a Chromo4TM system (BioRad, Foster City, California) as described by the manufacturer. Primers for miR-16-5p quantitative RT-PCR were obtained from Qiagen, and RNU6 small nuclear RNA ((Exiqon, Denmark) was employed as endogenous control for data normalization. ΔΔCt Method was applied to perform data analysis.

### Statistical Analysis

All the presented statistical tests were performed, applying GraphPad Prism version 8.4.3 software. The statistical paired t-test was employed to compare the mean of 3 paired biological repeats, and significant differences are defined as *P* < 0.05.

## RESULTS

### miR-16-5p is proposed to target WIP1protein affecting p53 pathway

The miRTarBase experimentally validated microRNA-target interactions database confirmed *PPM1D* as a target for miR-16-5p with strong evidence (Reporter assay, qRT-PCR and Western blot). In terms of miRDB target prediction database, miR-16-5p was at the top 20 miRNAs targeting *PPM1D* expression.

**Figure 1:**
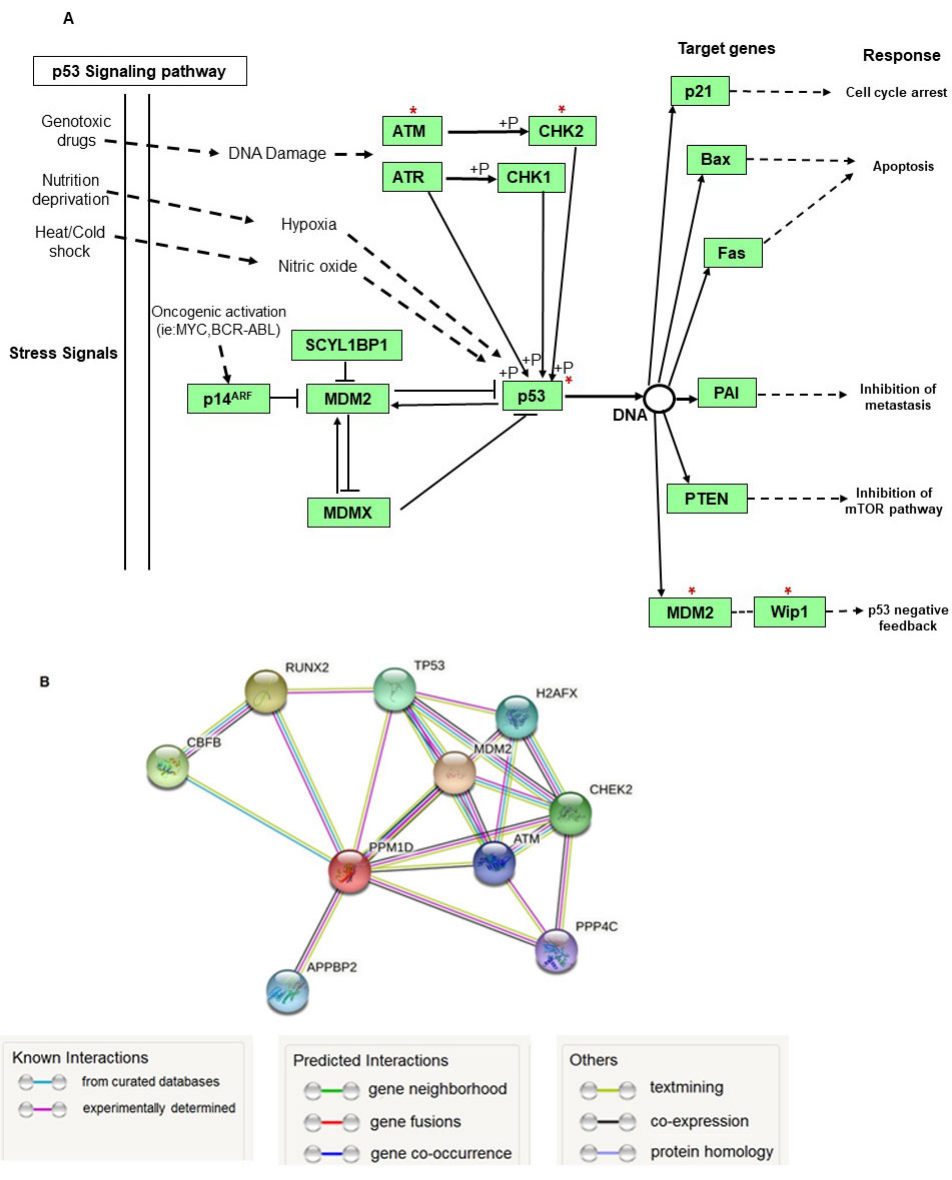
The importance of WIP1 in the p53 signaling pathway and its Protein–protein interaction network. (A) The p53 pathway map (map04115) was provided using the KEGG database. The red stars above each gene represent the genes whose activity is affected by WIP1 via dephosphorylation; (B) WIP1 Protein-protein interaction network was visualized by STRING with high confidence (0.7). The edges represent protein-protein associations which are meant to be specific and meaningful. This does not necessarily mean they are physically binding to each other. MDM2: Mouse Double Minute 2 Homolog; WIP1: Wild-type p53-Induced Phosphatase 1.

KEGG p53 pathway has shown Wip1 protein as one of the main negative regulators of p53 activity. In addition, Wip1 activates the main negative regulator of p53, Mdm2, and inactivates the kinases which phosphorylate and enhance p53 activity including CHK2 and ATM [Figure 1A]. Furthermore, STRING database confirmed the functional association between Wip1 and p53 and other proteins involved in p53 activity with a high confidence (0.7 score interaction) [Figure 1B].

### The cytotoxic effect of RG7388 on ALL cell lines and primary cultures

**Figure 2:**
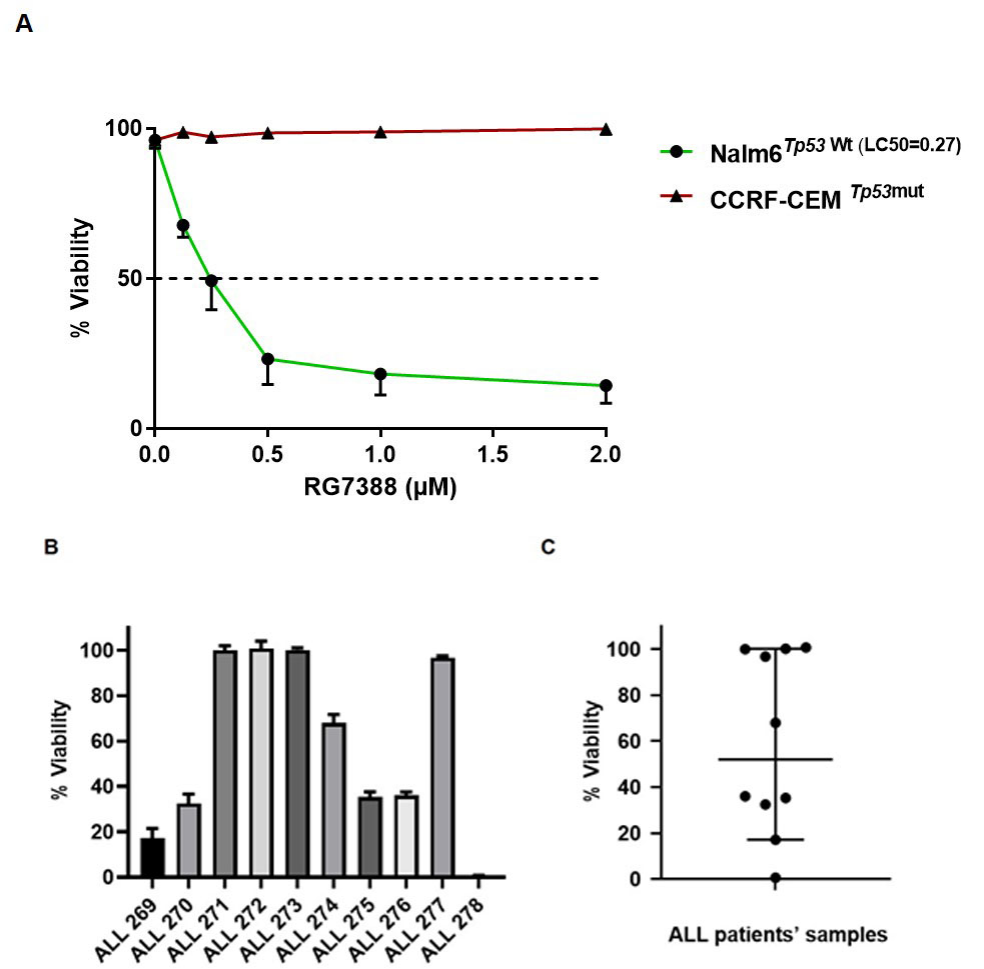
The sensitivity to MDM2 antagonists RG7388 in ALL cell lines and childhood ALL primary cells. (A) Wild-type *TP53* Nalm6 cell line is significantly more sensitive to growth inhibition by RG7388 treatment compared to mutant *TP53* CCRF-CEM cell line; (B) 10 pediatric ALL samples exposed to RG7388 (0.5 μM) for 72 h. RG7388 markedly decreased cell viability in most samples while assessed by MTT assay; (C) dot-plot of % viability for 10 pediatric ALL samples exposed to RG7388 (0.5 μM) for 72 h. Data shown are the average of three independent experiments and error bars represent SEM. ALL**:** Acute lymphoblastic leukemia.

The subsequent experiments were carried out to evaluate the sensitivity of two established ALL cell lines and ALL primary cells to RG7388, and the influence of ectopic overexpression of miR-16-5p on this sensitivity. The cytotoxicity of RG7388 and its p53-dependent effect was investigated using the MTT assay on Nalm6 wild-type *TP53* and CCRF-CEM mutant *TP53* cell lines. The LC_50_ values (Lethal Concentration 50%) indicated that wild-type *TP53* Nalm6 cell line was significantly more sensitive to RG7388 (0.27 ± 0.05 (SEM) μM) compared to CCRF-CEM mutant *TP53* cell line (> 2 μM) [Figure 2A]. The cytotoxic effect of RG7388 was also investigated on the cell viability of primary cultures generated from materials donated by ALL patients [Table 1]. 10 ALL samples were incubated with DMSO (0.5%) as control and RG7388 (0.5 μM), and they were examined for viability after 72 h using the MTT assay. RG7388 induced a cytotoxic effect on ALL cells, and 60% of those (6 out of 10) were sensitive and 40% (4 out of 10) were resistant to RG7388 with the delivered dose [Figure 2B]. RG7388 led to a significant reduction in the viability of sensitive ALL primary cells compared to their untreated counterpart (*P* < 0.05 or *P* < 0.001), with ALL 278 sample as the most sensitive sample (% viability = 0.66% ± 0.04, *P* = 0.0005). Overall, the median % survival for primary cultures was 53% [Figure 2C]. Notably, 3 out of 4 RG7388 resistant samples (75%) relapsed [Table 1].

**Table 1:** Clinicopathological data for 10 samples of pediatric ALL

Patient's number/Variable*	**Sex** **Age (years)**	**Peripheral blood/Bone marrow**	***De novo*/** **Relapsed**	**Pre-B cell/** **T cell**	Cytogenetics**	**Response** **to RG7388**
ALL 269	Boy (2)	Peripheral blood	*De novo*	Pre-B cell	No	Sensitive
ALL 270	Girl (2)	Bone marrow	*De novo*	Pre-B cell	No	Sensitive
ALL 271	Boy (3)	Bone marrow	*De novo*	Pre-B cell	No	Resistant
ALL 272	Girl (5)	Peripheral blood	Relapsed	Pre-B cell	No	Resistant
ALL 273	Boy (9)	Bone marrow	Relapsed	T cell	No	Resistant
ALL 274	Boy (6)	Bone marrow	*De novo*	Pre-B cell	T (12, 21)/ETV6-RUNX1	Sensitive
ALL 275	Girl (3)	Peripheral blood	*De novo*	Pre-B cell	T (1, 19)/TCF3-PBX1	Sensitive
ALL 276	Girl (5)	Peripheral blood	*De novo*	Pre-B cell	No	Sensitive
ALL 277	Girl (5)	Peripheral blood	Relapsed	T cell	No	Resistant
ALL 278	Boy (12)	Peripheral blood	*De novo*	Pre-B cell	No	Sensitive

*The blast proportion for all patients’ samples was over 70%. **The most common cytogenetics (T (4; 11)/KMT2A-AFF1, T (9; 22)/BCR-ABL1, T (1; 19)/ TCF3-PBX1, T (12; 21)/ ETV6-RUNX1) were only analyzed.

### Functional activation of the p53 pathway in ALL cell lines and primary cultures in response to RG7388

**Figure 3:**
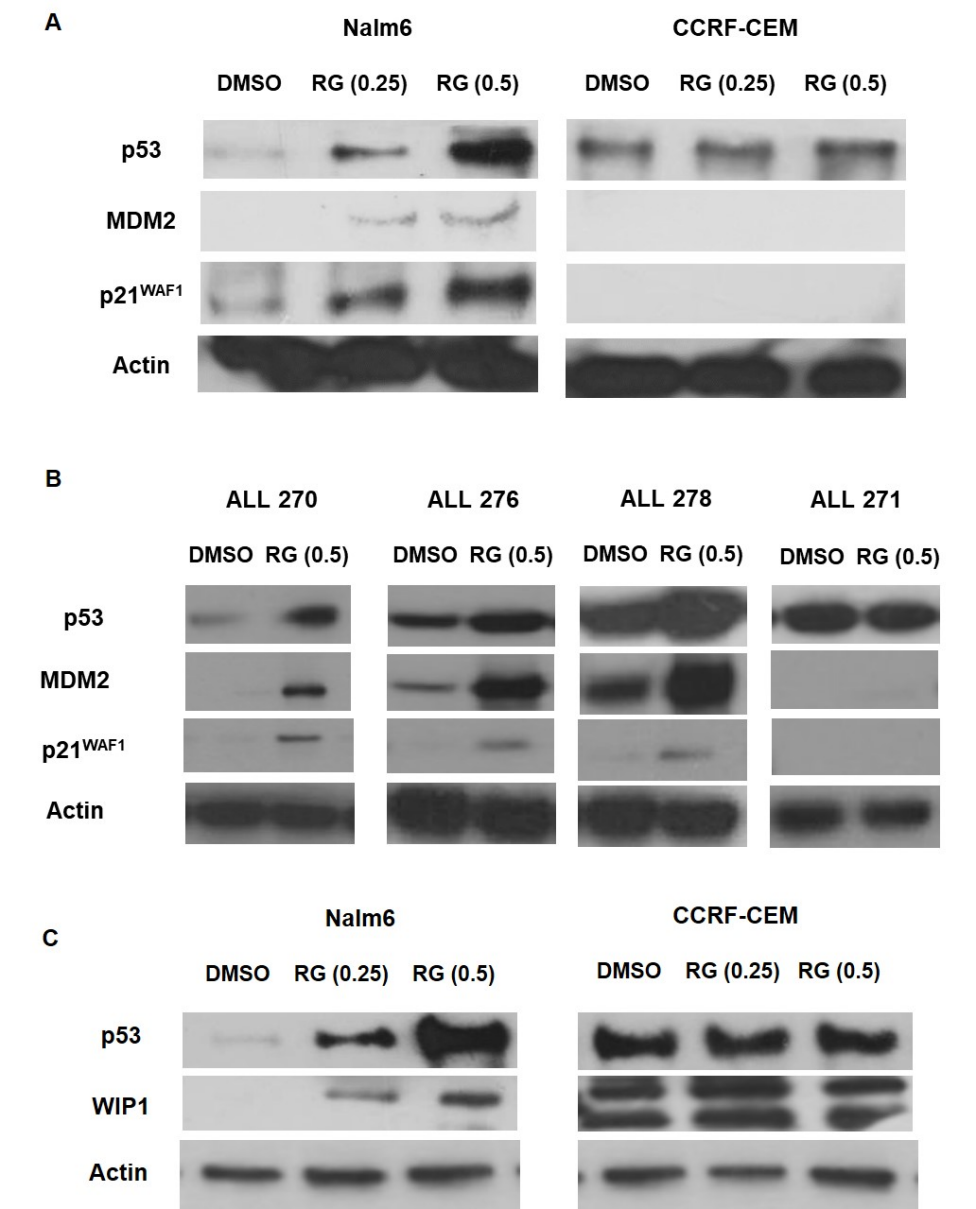
p53 functional stabilization in pediatric ALL cells in response to RG7388. Western blot analysis for (A) established Nalm6 and CCREF-CEM cell lines and (B) representative of patient samples with functional p53 and dysfunctional p53. RG7388 showed stabilization of p53 and upregulation of p53 transcriptional target gene protein levels, MDM2 and p21^WAF1^, 6 h after the commencement of treatment in wild-type *TP53* Nalm6 and p53 functional ALL cells with the indicated doses (μM). However, it had no effect on downstream transcriptional targets of p53 in mutant *TP53* CCRF-CEM and dysfunctional p53 patient samples with the delivered dose of RG7388 (μM); (C) western blot analysis indicated stabilization of p53 and induction of WIP1 expression following 6 h treatment with indicated doses of RG7388 (μM) in wild-type *TP53* Nalm6 in a concentration manner. Conversely, there was no effect on the p53 stabilization and WIP1 expression in mutant *TP53* CCRF-CEM with the delivered dose range of RG7388 (μM). RG, RG7388. ALL: Acute lymphoblastic leukemia; MDM2: mouse double minute 2 Homolog; WIP1: wild-type p53-induced phosphatase 1.

Functional assessment of the p53 pathway was evaluated by measuring p53 induction and its stabilization following six hours’ exposure to RG7388, and consequent overexpression of its downstream targets including Mdm2 and p21^WAF1^ proteins by Western blot. The p53-dependent response to RG7388 showed that RG7388 elevated p53 stabilization and overexpression of p21^WAF1^ and Mdm2 protein levels 6 h after the commencement of treatment in a concentration-dependent manner, and confirmed functional activation of wild-type *TP53* Nalm6 cell line by release from Mdm2. However, as anticipated, it had no impact on the expression of p53-dependent genes in the *TP53*-mutant CCRF-CEM cell line with the delivered dose range of RG7388 [Figure 3A and Supplementary Figure 1].

For the primary cultures with sufficient amount of protein lysates for western blot, there was consistency between MTT assay results and p53 function. RG7388 induced functional stabilization of p53 and expression of its downstream target genes, p21^WAF1^ and MDM2, in ALL samples that showed a significant decrease in their viability rate following treatment with RG7388. Conversely, there was no stabilization of p53 and induction of its downstream targets in primary cultures that were resistant to RG7388 [Figure 3B and Supplementary Figure 2].

### RG7388 induces Wip1 expression in a p53-dependent manner

The basal protein levels of Wip1 and its expression levels following treatment with RG7388 at the 1 × and 2 × LC_50_ value for Nalm6 were determined in both Nalm6 and CCRF-CEM cell lines [Figure 3C and Supplementary Figure 3]. RG7388 increased stabilization of p53 protein, with subsequent increased expression of Wip1 protein in Nalm6 cells in a concentration-dependent manner. However, RG7388 failed to stabilize p53 and induce Wip1 in CCRF-CEM cells, indicating p53-dependent expression of Wip1 protein. Notably, both full-length (FL-WIP1) and its previously described shorter isoform (S-WIP1) of Wip1 protein were expressed by CCRF-CEM cell line.

### miR-16-5p negatively regulates WIP1 expression and sensitizes Nalm6 cells to RG7388

**Figure 4:**
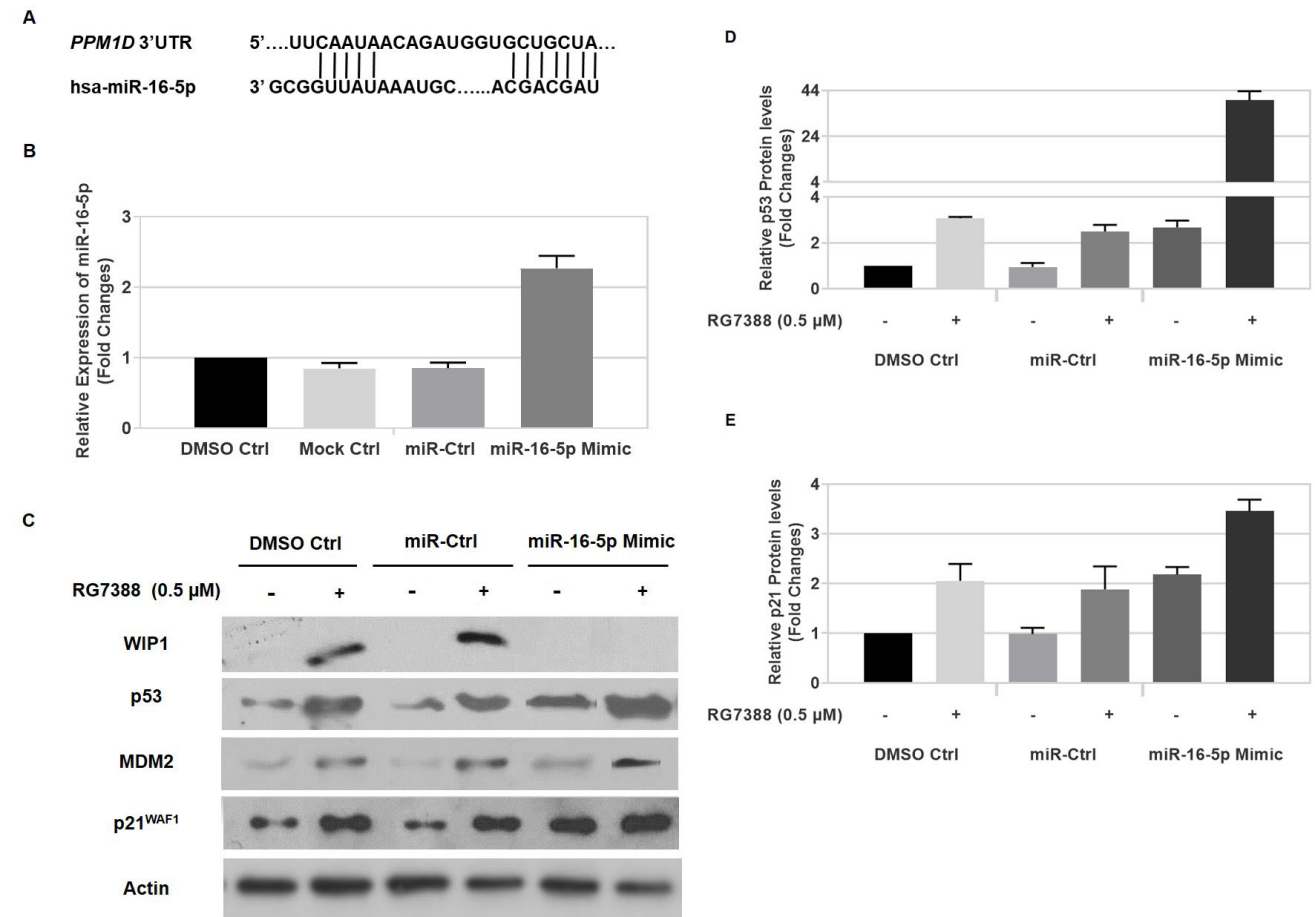
miR-16-5p suppresses WIP1 expression, affects p53 stabilization and its activity, and enhances sensitivity to RG7388. (A) miR-16-5p sequences and its putative binding sites in the 3′-UTR of PPM1D; (B) Nalm6 cells were transfected with negative control miRNA and miR-16-5p mimic (200nM). Total RNA was extracted from DMSO (0.5%) treated Nalm6, mock control, and those transfected with negative control miRNA or miR-16-5p mimic at 48 h after transfection. miR-16-5p levels were measured by quantitative RT-PCR shown significant ectopic overexpression of miR-16-5p in transfected cells with miR-16-5p compared to DMSO control, mock control and negative control miRNA; (C) ectopic overexpression of miR-16-5p suppresses WIP1 expression, enhances p53 stabilization and upregulates expression of p53 target genes, p21^WAF1^ and MDM2 in wild-type *TP53* Nalm6 treated with RG7388. Nalm6 cells were transfected with miR-16-5p (200nM) or scrambled miRNA. Cells were treated with RG7388 (0.5 μM) at 48 h after transfection and their protein levels analyzed at 6 h after RG7388 treatment. The intensity of p53 blots (*P* < 0.05 or *P* < 0.1) (D) and p21^WAF1^ blots (*P* < 0.05) (E) was quantified by Image J software and normalized with the DMSO control. Data shown are the average of three independent experiments and error bars represent SEM. Ctrl, Control. *P < 0.05, **P < 0.01

The p53-Wip1 autoregulatory feedback loop regulates both expression levels of *PPM1D* gene and p53 activity. Previous studies showed that post-transcriptional regulation influences the expression of Wip1 protein and confirmed that miR-16-5p inhibits WIP1 expression through targeting 3’UTR of WIP1 [Figure 4A]. Thus, it is expected that miR-16-5p affects the response to RG7388 through regulating WIP1 in a p53-dependent manner.

To investigate the hypothesis, firstly, Nalm6 cells were transfected with miR-16-5p mimic or scrambled miRNA oligonucleotides as a negative control. Real-time PCR results confirmed high expression of miR-16-5p in Nalm6 cells resulting from the transfection of miR-16-5p mimic after 48 h. A significant increase was observed in the ectopic expression of miR-16-5p (by ~ 2.5-fold) compared with DMSO control, mock control, and negative control (*P* < 0.05) [Figure 4B].

We evaluated the protein levels of Wip1, p53, p21^WAF1^ and Mdm2 in Nalm6 treated with RG7388 in the presence of miR-16-5p mimic and negative control in order to investigate the impact of altered levels of miR-16-5p on the expression of the proteins. The high expression of miR-16-5p in Nalm6 led to a significant increase in p53 protein levels (by ~ 3.0 fold) [Figure 4C and D], p21^WAF1^ protein levels (by ~ 2.5 fold) [Figure 4C and E] and Mdm2 [Figure 4C] compared to Nalm6 transfected with miRNA oligonucleotides as a negative control and DMSO control (*P* < 0.05) irrespective of treatment with RG7388. Interestingly, ectopic overexpression of miR-16-5p significantly suppressed induction of WIP1 in Nalm6 treated with RG7388, while scrambled negative control had no effect on the expression levels of WIP1 after RG7388 treatment. Furthermore, overexpression of miR-16-5p caused a significant upregulation of p53 (by ~ 15.5-fold, *P* < 0.01) [Figure 4C and D], and its target genes, p21^WAF1^ (by ~ 2-fold, *P* < 0.05) [Figure 4C and E] and Mdm2, in Nalm6 treated with RG7388 compared to negative control treated with RG7388. These results clearly demonstrated that miR-16-5p negatively regulates Wip1 protein levels, which consequently affects the p53 pathway and response to RG7388.

## DISCUSSION

Acute lymphoblastic leukemia (ALL) is the most prevalent type of pediatric blood cancer in which *TP53* mutations are infrequent, less than 5%, at diagnosis but rise to about 10% in relapsed ALL^[24]^. Although complete remission (CR) is achieved for many patients at the end of the induction phase of treatment, relapse and drug resistance are major challenges in treating cancer^[25]^. Recently, p53-Mdm2 binding antagonists have been advanced to restore wild-type p53 function with subsequent induction of cell cycle arrest and apoptosis. These targeted therapeutic agents have shown *in vitro* promising results alone and in combined treatments^[26-31]^, and encouraging clinical trial outcomes in diverse types of cancer including blood malignancies^[8,32-35]^. miR-16-5p was previously reported as a post-transcriptional regulator of Wip1 in some types of cancer^[13]^. miRTarBase and miRDB microRNA-target interactions databases, KEGG pathway and STRING databases used in this study indicated the critical role of miR-16-5p in p53 activity through targeting PPM1D expression in ALL. Interestingly, the STRING database clearly showed strong interactions between Wip1 and p53 or its regulators comprising ATM, Mdm2 and CHK2 proteins^[20]^.

The present study evaluated, for the first time, the impact of the Mdm2-p53 binding antagonist RG7388 in ALL cell lines, and primary cultures generated from materials donated by ALL patients. Moreover, this is the first study elucidating the positive effect of ectopic miR-16-5p on increasing sensitivity to RG7388 in *TP53* wild-type leukemic cells.

Among the individual cell lines studied, wild-type *TP53* Nalm6 cell line was significantly more sensitive to RG7388 compared to mutant *TP53* CCRF-CEM cell line, which is in line with its mechanism of action^[35]^. These results partially confirm previous limited previous studies that indicated a significant decrease in the cell viability of wild-type *TP53* ALL cell lines following treatment with Mdm2 inhibitor Nutlin-3a^[36,37]^.

Within a panel of primary cultures, RG7388 significantly decreased the viability of most ALL cells (6 out of 7, 86%) isolated from *de novo* patients. Conversely, primary cultures derived from relapsed patients were resistant to the cytotoxicity effect of RG7388 at the delivered dose. Given the fact that response to RG7388 is dependent on wild-type p53, resistance to RG7388 could be related to *TP53* mutations that are infrequent in childhood ALL patients at diagnosis, but they increase at relapse. Since the genomic status of *TP53* gene is the major determinant of response to Mdm2 inhibitors, DNA sequencing of *TP53*, as the gold standard method, is highly recommended for identification of *TP53* mutations in primary cells, particularly in the personalized directed use of inhibitors such as RG7388^[38]^. It is also possible that amplification/overexpression of WIP1 in different types of cancer including hematological tumors^[10]^ results in dysfunctional wild-type *TP53* and resistance to p53-dependent treatments comprising Mdm2 inhibitors^[11]^.

In accord with the action mechanism of Mdm2 inhibitors, RG7388 treatment resulted in more stabilization of p53 and increased expression of its downstream targets, p21^WAF1^ and Mdm2 in wild-type *TP53* Nalm6, in a concentration-dependent manner, and functional p53 primary cultures at the delivered dose of RG7388. In contrast, no significant enhancement of p53 downstream target genes was observed in mutant *TP53* CCRF-CEM and non-functional p53 ALL samples following treatment with RG7388. These outcomes are consistent with other studies that reported induction of the p53 pathway in wild-type *TP53* ALL cell lines after treatment with Nutlin-3a^[36,37]^, CLL patients treated with RG7388^[28]^, and Acute myeloid leukemia patients’ clinical response to RG7388^[39]^.

Given the promising phase 1 results observed in blood cancer patients treated with Mdm2 inhibitors RG73122^[40]^, RG7388^[8,39]^, and AMG-32^[5]^, and the prognostic value of miR-16 expression in childhood ALL^[41]^ and CLL^[15]^, we evaluated the impact of miR-16-5p expression on the induction of p53 pathway and response to Mdm2 inhibitor RG7388.

Wip1 protein levels were measured at the basal level and following treatment with RG7388 in both Nalm6 and CCRF-CEM cell lines. In comparison with CCRF-CEM, in which WIP1 is highly expressed at the basal levels, WIP1 is not detectable at its basal levels in Nalm6. As expected, RG7388 treatment led to more stabilization of p53 and its target, WIP1, in a concentration-dependent manner in wild-type *TP53* Nalm6. However, there was no stabilization of p53 and no upregulation of WIP1 in *TP53* mutant CCRF-CEM cell line, demonstrating that the effect is p53-dependent. Notably, CCRF-CEM harbors a heterozygous *PPM1D* mutation (c.1327A>G; p.N443D) reported by the COSMIC (Catalogue of Somatic Mutations in Cancer)^[42]^.

It was also shown that upregulation of miR-16-5p (by ~ 2.5-fold) significantly induced p53 stabilization (by ~ 3-fold) and upregulated its downstream target p21^WAF1^ (by ~ 2.5-fold). p53 is considered as a representative haploinsufficient tumor suppressor gene in which a small change in its protein levels and/or its activity could immensely affect tumourigenesis in both mice and humans^[43]^. Interestingly, ectopic overexpression of miR-16-5p dramatically suppressed WIP1 expression after treatment with RG7388, followed by a significant rise in the p53 stabilization (by ~ 15.5-fold) and p21^WAF1^ upregulation (by ~ 2-fold). These results were in accord with several studies indicating the therapeutic impact of combined treatment between Wip1 inhibitor, GSK2830371, and Mdm2 inhibitors for increasing sensitivity to p53-Mdm2 antagonists in wild-type *TP53* cell lines^[11,31,44-46]^. Mdm2 inhibitors release p53 from its negative regulator, Mdm2, which results in more stabilization of p53. Inhibition of Wip1 leads to increased phosphorylated p53 at serine 15 which activates the p53 pathway playing a critical role in cell cycle arrest mostly via upregulating of p21^WAF1^, and apoptosis through upregulating proapoptotic genes including *PUMA*^[11,31,46]^, and downregulating antiapoptotic genes particularly, *BCL2* and *BIRC5* (survivin)^[46,47]^. Furthermore, miR-16-5p targets multiple cell cycle genes simultaneously, which leads to the accumulation of cells in G0/G1^[47-49]^ and directly targets the antiapoptotic *BCL2* gene which results in enhancing apoptosis^[50,51]^ and modulating multidrug resistance. Therefore, in addition to genomic status of *TP53* and its downstream target genes involved in apoptosis and cell cycle arrest, the expression levels of miR-16-5p might be considered as a marker to predict the sensitivity to Mdm2 inhibitors like RG7388 and other drugs working through the p53 pathway^[52]^. It is of note that local delivery, which is limited to the localized primary tumors, systemic route, viral delivery, and non-viral administration of miRNAs, are choices in delivering miRNAs^[53]^.

In conclusion, the current study indicated the cytotoxic effect of the Mdm2 inhibitor, RG7388, on ALL patients’ primary cells in a p53-dependent manner. Moreover, it was shown that ectopic overexpression of mirR-16-5p increases sensitivity to RG7388 in ALL cells by suppressing WIP1 expression and inducing p53 stabilization. These data indicated, for the first time, the mechanistic importance of miR-16-5p in the pathophysiology of ALL, sensitivity to RG7388, and suggested its combination with RG7388 as a novel strategy for therapeutic targeting of non-mutant p53 pediatric ALL patients.

## DECLARATIONS

### Availability of data and materials

All data generated or analyzed during the current study are available from the corresponding author upon reasonable request.

### Copyright

© The Author(s) 2023.
