# Supplementary material for: miR-16-5p enhances sensitivity to RG7388 through targeting PPM1D expression (WIP1) in Childhood Acute Lymphoblastic Leukemia
Source: Cancer Drug Resist. 2023 Apr 29;6(2):242–56. doi: 10.20517/cdr.2022.113 (PMC10344722; doi:10.20517/cdr.2022.113)
Supplement: Supplementary file 1 [file cdr-6-2-242-SupplementaryMaterials.pdf]

Supplementary Figure 1

Actin: From right to left in order:
Nalm6 DSMO, RG (0.250, RG (0.5);
CCRF-CEM DSMO, RG (0.250, RG (0.5);

p21: From right to left in order:
CCRF-CEM DSMO, RG (0.250, RG (0.5);
Nalm6 DSMO, RG (0.250, RG (0.5)

p53: From right to left in order:
Nalm6 DSMO, RG (0.250, RG (0.5);
CCRF-CEM DSMO, RG (0.250, RG (0.5);

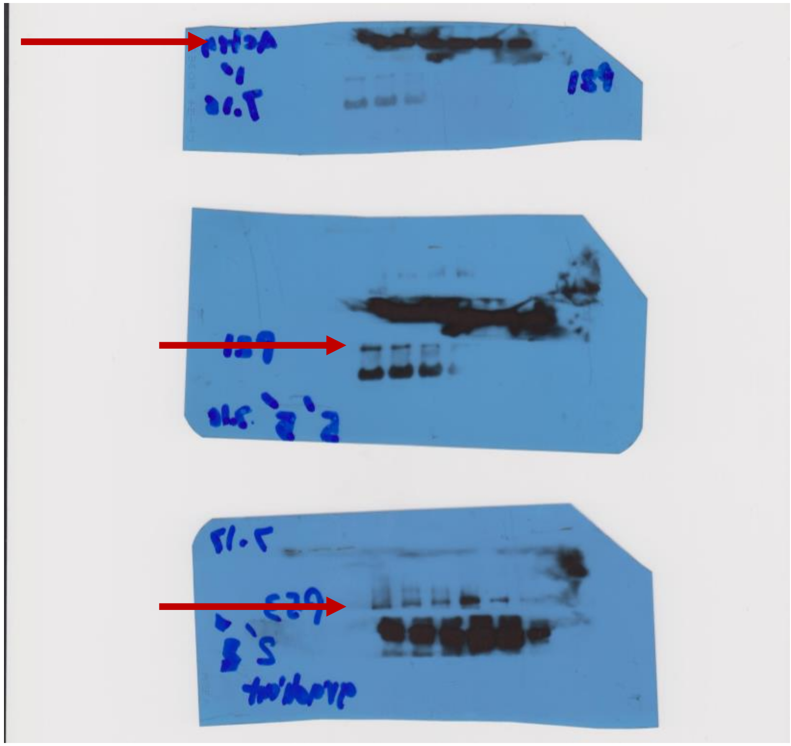

Mdm2: From left to right in order:
Nalm6 DSMO, RG (0.250, RG (0.5);
CCRF-CEM DSMO, RG (0.250, RG (0.5)

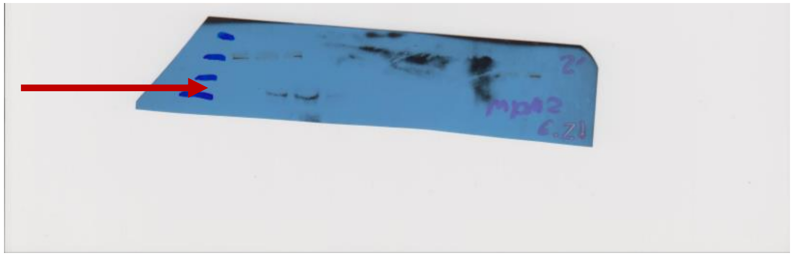

Original results of western blot for actin, p21, p53, and Mdm2 proteins in regard to the Figure 3A

Supplementary Figure 2

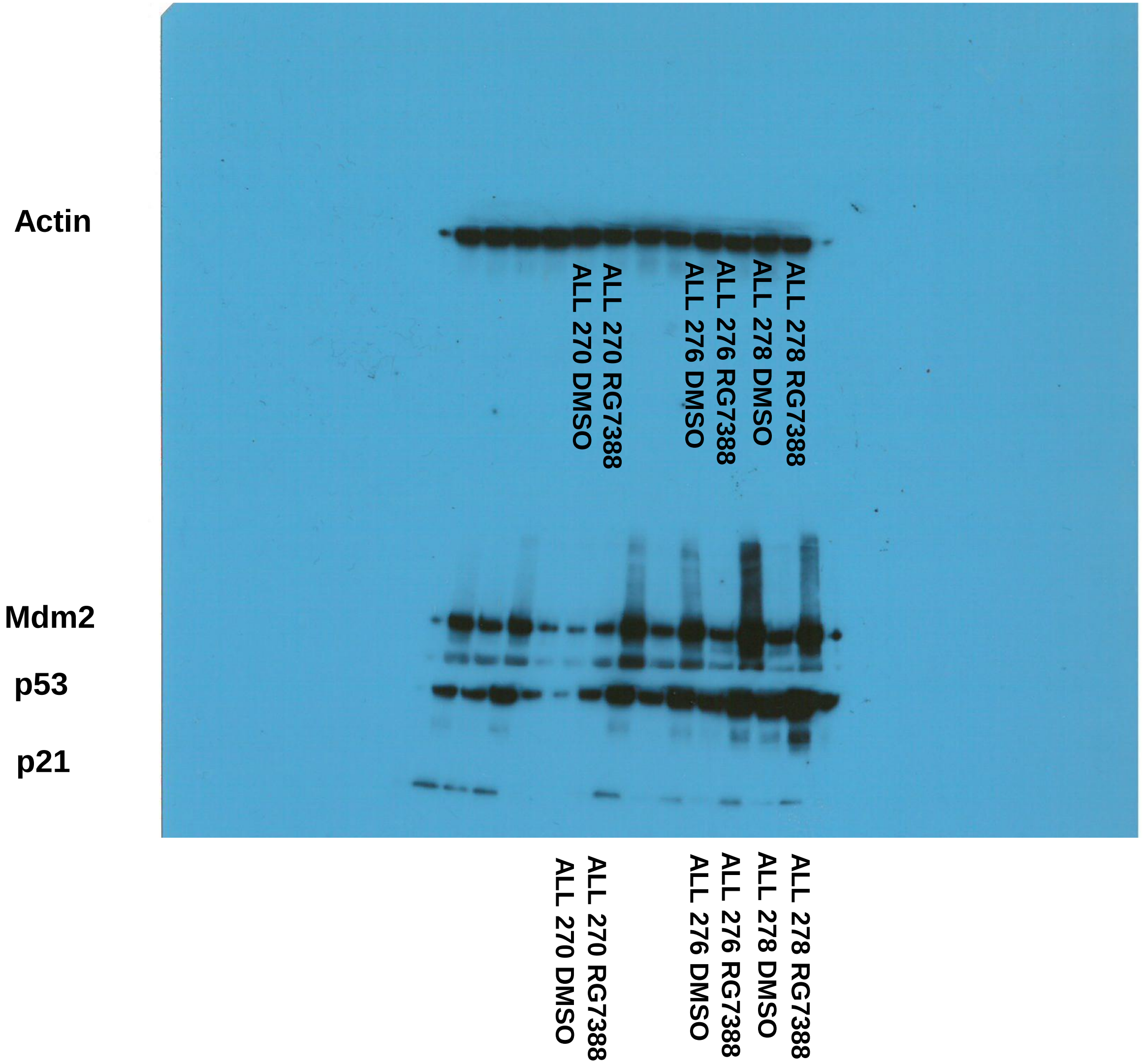

Original results of western blot for actin, p21, p53, and Mdm2 proteins in regard to the Figure 3B

Supplementary Figure 3

p53 from left to right in order:
Nalm6 DSMO, RG (0.250, RG (0.5);
CCRF-CEM DSMO, RG (0.250, RG (0.5)

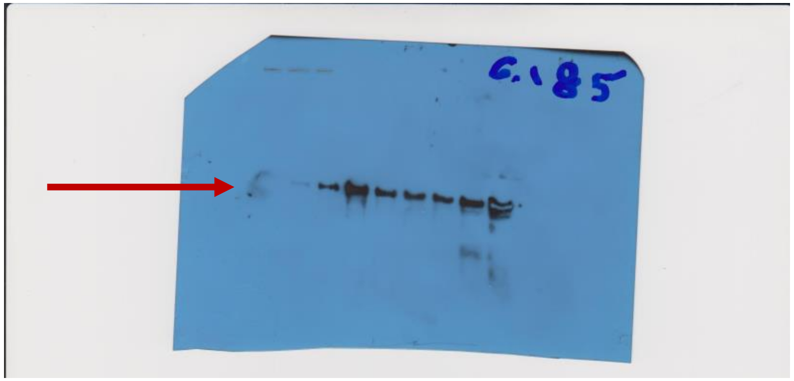

WIP1 and actin from left to right:
In order: Nalm6 DSMO, RG (0.250, RG (0.5);
CCRF-CEM DSMO, RG (0.250, RG (0.5)

The same as above with different exposure time.

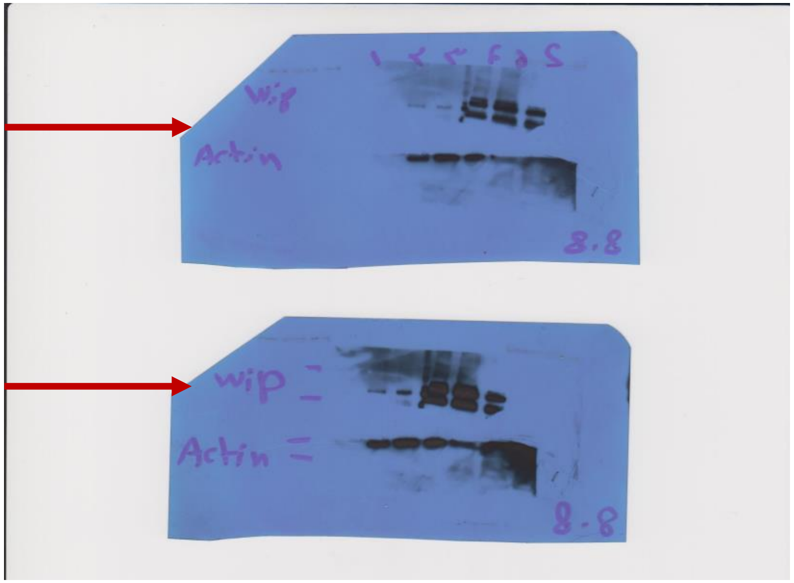

Original results of western blot for actin, p53, and Wip1 proteins in regard to the Figure 3C
